# 

**Published:** 1850-08

**Authors:** 


### CONTENTS

OF THE

### MEDICAL EXAMINER.

NEW SERIES—NO. LXVIII.—AUGUST, 1850.

### ORIGINAL COMMUNICATIONS.

On Acclimation. An Inaugural Essay presented for the Degree of Doctor of Medicine in the University of Pennsylvania. By Philip C. Williams, M. D., of Winchester, Virginia, - - 439

Nitrate of Silver in Epidemic Dysentery. By Lew. Slusser, M. D., of Canal Fulton, Ohio, - - - - - 450

Case of Arrested Muscular Development. By P. K. Huntington, M. D., of Perry, Wyoming county, New York. (Communicated by Prof. J. K. Mitchell), - - - - - - 453

Case of Lactation in a Male. By C. W. Hornor, M. D., of Philadelphia. (Communicated by Prof. Dunglison), - - - 454

### BIBLIOGRAPHICAL NOTICES.

Southern Medical Reports: Consisting of General and Special Reports of the Medical Topography, Meteorology and Prevalent Diseases in the following States: Louisiana, Alabama, Mississippi,

North Carolina, South Carolina, Georgia, Florida, Arkansas, Tennessee and Texas; to be published annually. Edited by E. D. Fenner, M. D., of New Orleans, Member of the American Medical Association, &c. &c. (Concluded), - - 455

Essays on the Puerperal Fever, and other diseases peculiar to Women; selected from the writings of British Authors previous to the close of the eighteenth century. Edited by Fleetwood Churchill, M. D., M. R. I. A., &c. &c., - - - 469

### EDITORIAL.

Assimilated Rank, - - - - - - - - 483

Resignation of Prof. Dudley, - - - - - - 484

Visit of Liebig, - - - - - - - 484

Resignation of Prof. Mott, - - - - - - 485

Baltimore Medical and Surgical Journal, - - - - 485

Removal of an Ovarian tumor, - - - - - 485

Medical Faculty of the University of New York, - - - 485

Homœopathy condemned by the law, - - - - 485

Death of the President of the United States, - - - - 486

Maclise's Surgical Anatomy, - - - - - - 487

Appointment of Dr. T. O. Edwards, - - - - - 487

Death of Dr. John T. Shotwell, - - - - - 487

Deaths in Philadelphia from June 22d to July 20th, 1850. Reported by Mr. James Aitken Meigs, Student of Medicine, - - 488

### RECORD OF MEDICAL SCIENCE.

### SURGERY.

Self-inflicted wound of the throat, laying open the Œsophagus—Recovery, - - - - - - - - - 489

Strabismus.—Division of the Rectus by means of Lane's Knife made by Savigny, - - - - - - - - 492

### OBSTETRICS.

Case of Pregnancy, notwithstanding previous severe injury to the organs concerned in Childbirth, - - - - - 492

PATHOLOGY AND PRACTICE OF MEDICINE.

Continued success of the Kousso in promoting the Expulsion of the Tape-worm, - - - - - - - - 494
Case of "White Blood," - - - - - - - 495

CHEMISTRY.

On the changes which Ether, Alcohol, and bodies of a similar constitution, suffer, when taken into the Circulation. By Charles W. Wright, M. D., of Cincinnati, - - - - 496

MATERIA MEDICA AND THERAPEUTICS.

Method of depriving Quinine of its Bitterness, - - - 498

